# Successful insertion and expression of a tetracycline transactivator in Anopheles stephensi associated with increased egg production and decreased hatching rate

**DOI:** 10.21203/rs.3.rs-6270709/v1

**Published:** 2025-04-16

**Authors:** Ehud Inbar, Ishaan Samantray, Robert T. Alford, Robert A. Harrell, Grace Jennings, Tales V. Pascini, Tint T. Wai, Abraham Eappen, Stephen L. Hoffman, Peter F. Billingsley

**Affiliations:** Sanaria Inc; Sanaria Inc; University of Maryland; University of Maryland; Sanaria Inc; Sanaria Inc; Sanaria Inc; Sanaria Inc; Sanaria Inc; The Vital Narrative

**Keywords:** Anopheles, driver line, Tetracycline, rtTA

## Abstract

Sanaria^®^ has pioneered production of aseptic, purified, vialed cryopreserved *Plasmodium falciparum* (Pf) sporozoites (SPZ) as vaccines and for controlled human malaria infections. More than 3,500 individuals have received more than 9,700 injections of PfSPZ, worldwide. The PfSPZ are manufactured in aseptically reared female *Anopheles stephensi* mosquitoes. Since PfSPZ vaccines are intended primarily for some of the most disadvantaged people in the world, keeping costs low is imperative. One approach to reducing cost of goods is to eliminate male mosquitoes from the production process, thereby doubling the numbers of PfSPZ-producing mosquitoes per unit space. We intend to do this by creating *A. stephensi* with a male-lethal allele controlled by the tetracycline conditional gene expression system. Herein, we report the first step in this process, the creation of a driver line that expresses the reverse tetracycline transactivator (rtTA). After sub-optimal results using the bZip early embryonic promoter, we produced 3 mosquito driver lines that expressed rtTA from 3 different genomic loci under the early embryonic vasa promoter. Expressing the rtTA under the vasa promoter significantly increased rtTA mRNA levels compared to its level under bZip. We were unable to achieve homozygosity in two of these lines even after 26 generations. In the third line, the insertion was in an intron of a putative juvenile hormone diol kinase gene. Homozygosity was reached in this line after passage through 7 generations, indicating that the inserted vasa-rtTA nucleotides did not interfere with the function of an essential genomic locus. rtTA mRNA expression levels were higher than in the other two lines, supporting the hypothesis that failure to achieve homozygosity was not due to rtTA expression but to insertion position. The homozygous line produced ~ 18% more eggs per female and a hatching rate of larvae from eggs was 39% lower than of wild-type *A. stephensi*. The next step will be to cross the driver line with an effector line containing a male-linked lethal gene regulated by the tetracycline responsive element (TRE).

## Background

Vector-borne diseases have a devastating impact worldwide, transmitting viruses, bacteria and parasites which account for 17% of all infectious diseases with more than 700,000 death annually (1). Malaria is caused by *Plasmodium* parasites which are transmitted by anopheline mosquitoes. In 2023, malaria caused 263 million illnesses and 597,000 deaths, mainly in young children (1). The dengue virus is transmitted by *Aedes* mosquitoes which poses a risk for roughly 4 billion people in 132 countries with annual mortality rate of 40,000 individuals (1).

Sanaria’s *Plasmodium falciparum* (Pf) sporozoites (SPZ) products are composed of live, aseptic, purified cryopreserved PfSPZ (2). More than 3,500 individuals have received more than 9,700 doses of PfSPZ products in 44 clinical trials in 14 countries in North America, Europe, Africa, and Asia (3–31). Sanaria^®^ PfSPZ vaccines are safe and well tolerated and have achieved 100% efficacy against heterologous controlled human malaria infection at 12 weeks after last dose of vaccine (29), sustained protection for at least two years against field transmitted malaria in Africa, including 57% protection against Pf infection for two years with no boosting in Mali (32), and protection for at least 6 months against highly variant Pf parasites in Papua (Indonesian West New Guinea) (33). In 2025, clinical trials will begin of Sanaria’s 3rd generation vaccine, genetically attenuated PfSPZ-LARC2 Vaccine (34). Recent research conducted in the Netherlands has demonstrated that a genetically attenuated vaccine strain with just a single deletion of the mei2 gene (GA2) exhibits a vaccine efficacy of approximately 90% and is well-tolerated (35, 36). Given that PfSPZ-LARC2 is a double knockout vaccine strain, with deletions in both mei2 and LINUP, we anticipate a similar or potentially greater protective efficacy, possibly achievable with just one dose.

All PfSPZ products are currently produced in aseptic *Anopheles stephensi* mosquitoes. Major drivers of the cost of vaccine are the incubator space and the personnel required for production and management of aseptic *A. stephensi*. Only female mosquitoes carry the PfSPZ used in PfSPZ vaccines. If males could be eliminated from the manufacturing process, we would be able to produce double the numbers of PfSPZ in the same space and with the same personnel, thereby significantly reducing the cost of goods.

The first step in creating “female only” *An. stephensi* mosquitoes was to generate a driver line for conditional expression. To do this we used a tetracycline-controlled transcription mechanism, one that is central to tetracycline resistance of gram-negative bacteria (37), and has been adapted to conditionally express genes in different eukaryotic systems, including mammals and insects (38–42). In this system, a gene of interest is regulated by the tetracycline response element (TRE), which consists of multiple units of the tetracycline operator (TetO). Transcription is initiated only when the tetracycline transactivator (tTA), a DNA-binding protein, binds to the TRE (38, 42, 43). There are two main mechanistic adaptations of this system, Tet-On and Tet-off. In the Tet-off system, the tTA is bound to tetracycline or its derivative doxycycline to prevent binding of the tTA to the TRE; when the drug is removed, the tTA binds to the TRE to initiate transcription of the gene of interest. Conversely, in the Tet-on system, the tTA is modified to a reverse tetracycline transactivator (rtTA) which binds to the TRE in the presence of doxycycline to initiate transcription. When the drug is removed the rtTA is released from the TRE and transcription stops (38, 42, 43).

The Tet system has been used in vector control approaches to decrease populations of various disease-transmitting vectors, using Release of Insects carrying a Dominant Lethal gene (RIDL). RIDL is an improved version of the classical release of sterile insect technique (SIT) (44). Male *Aedes aegypti* mosquitoes carrying lethal genes are released to mate with wild-type females and transmit the lethal genes to future generations. In the absence of tetracycline or doxycycline in the field, lethal genes are induced and the mosquitoes are killed, usually at the early larval stage (40, 41, 45–49). Currently, there is an extensive effort to use RIDL to reduce populations of *Ae. Aegypti* to fight the spread of arboviruses including Zika, Yellow Fever and Dengue (47, 50, 51).

Our plan is to use the tetracycline conditional expression system to drive expression of a male lethal gene thereby creating “female-only” aseptic mosquitoes for PfSPZ production. Here we report our successful generation of a homozygous driver line of mosquitoes expressing rtTA (Tet-On) to be used to drive expression of a male lethal gene.

## Methods

### Mosquito rearing

*Anopheles stephensi* SDA500 were reared at the Insect transformation facility (ITF) of the University of Maryland’s institute for Bioscience and Biotechnology Research (IBBR) in Rockville, MD, at 28°C, 75% humidity and 12h:12h light:dark cycle with 30-minute dawn and dusk periods.

### Construction of driver lines: construction of vasa-rtTA in PiggyBac vector and embryo injections

The reverse tetracycline transactivator (rtTA) gene, fused to VP16 transcription activation domain was synthesized by GeneScript company (Piscataway NJ, USA). The vasa promoter was amplified from *An. stephensi* genomic DNA. We assembled vasa-rtTA-SV40 using NEBuilder^®^ HiFi DNA Assembly Cloning Kit (NEB #E5520S). The entire construct was ligated into the *piggyBac* transposon vector containing mBanana gene under the 3Xp3 promoter (52) for easy identification of transgenic An. *stephensi*. The injections were done as described previously (53, 54). Briefly, Preblastodermal embryos were injected, 40–60 minutes post embryo collection, with an injection mix consisting of 150 ng/μL of *piggyBac* vector and 193 ng/μL of *piggyBac* transposase mRNA in halocarbon oil, as described (54)

### DNA work, Splinkerette mapping and RNA work

DNA was extracted from individual adult mosquitoes using DNeasy (Qiagen #69504) as described (55). Splinkerette mapping of transposable elements was done as described for *D. melanogaster* (56). Briefly, genomic DNA was digested by BstYI (NEB # R0523S) and the fragments ligated to a Splinkerette element on 5’ and 3’ ends. Fragments containing the insertions were amplified by two cycles of PCR, using the primers from both ends of the *PiggyBac* insertions to the Splinkerette at the ends of the fragments, as designed [60]. The PCR amplicons were sent for Sanger sequencing and the sequences sequences were aligned against the An. *Stephensi* SDA500 genome at VEuPathDB, VectorBase (https://vectorbase.org/vectorbase/app/). RNA was extracted as described (55), using Qiagen RNeasy mini kit (Qiagen #74106) according to the manufacturer instructions. Complimentary (c)DNAs were synthesized using the High-Capacity cDNA Reverse Transcription Kit (Thermo Fisher # 4368814) on extracted RNAs. Real-time PCR reactions were done on cDNAs using SensiFAST^™^ Real-Time PCR Kits (Bioline # BIO-82005). The ribosomal protein S7 was used as a house keeping control (55).

### Assessment of fecundity

Three days after blood feeding, females were placed individually in plastic *Drosophila* cylinders containing water and sealed with cotton balls (55). The mosquitoes were incubated overnight at > 70% humidity, 28°C. The next day, the mosquitoes were removed from the tubes and the eggs were counted. The larvae were counted in the tubes 1–2 days after egg laying. The larvae hatching rate was determined as the percent larvae per the total number of eggs in each tube. Data were analyzed using GraphPad Prism version 10.2.1 for Windows.

## Results

### Generation of An. stephensi driver line, expressing rtTA under the vasa promoter

**Figures 1:**
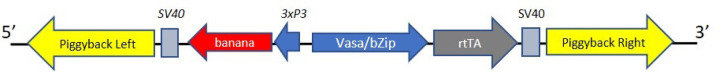
Schematic representation of the driver line construct. The rtTA ORF was designed to be expressed under the vasa or bZip promoters. The mBanana is expressed under the 3Xp3 promoter. The SV40 3’UTR is used as transcription termination for both genes. The cassette was placed between 2 *piggyBac* transposon elements (yellow)

We constructed a driver line in which the reverse tetracycline transactivator (rtTA, Tet-On) was expressed under the early embryonic promoter, bZip. Assessments of rtTA transcript abundance by quantitative (q) RT-PCR revealed very low mRNA levels of rtTA in eggs, ovaries and adults (data not shown). We therefore replaced the bZip promoter with the early embryonic vasa promoter. For this vasa-rtTA construct, an mBanana fluorescent marker, driven by the 3XP3 promoter was placed in the reverse orientation to the vasa-rtTA expression cassette. The construct was placed into a *piggybac* vector (52) between two transposon elements (Fig. 1), then mixed with a transposase-expressing vector (54) and injected into *An. stephensi* eggs at the early embryonic stage. Out of 854 eggs injected, 45 hatched and 29 (14 males and 15 females) survived to adults. Males (M) and females (F) were sorted and grouped into cages and crossed with the opposite sex of wild type mosquitoes. Larvae of the subsequent generation (G_1_), were screened using a fluorescent microscope and transgenic larvae expressing mBanana were collected. From the M group, 114 G_1_ larvae were mBanana-positive, of which 43 males and 39 females survived to adults. Fifty-six mBanana-positive larvae, 27 males and 29 females, were obtained from the F group, all surviving to adults. To avoid mixing of genotypes, we separated transgenic lines by collecting eggs from 20 individual G_1_ M-group females and 14 individual G_1_ F-group females. After oviposition, RNA was extracted from the G_1_ females and qRT-PCR used to assess rtTA mRNA abundance (data not shown).

### The rtTA transcription levels under early embryonic vasa and bZip promoters

**Figure 2:**
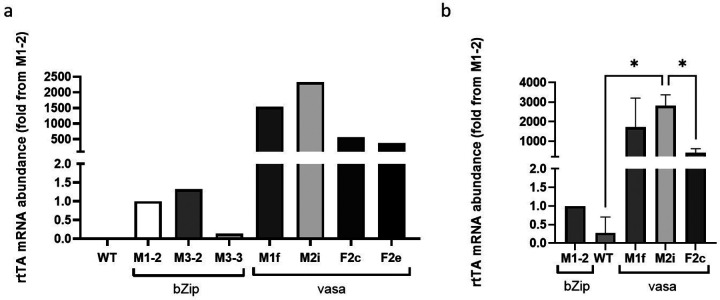
rtTA mRNA abundance under bZip and vasa early embryonic promoters. (a) Quantitative RT-PCR done on cDNAs produced from RNAs, extracted from individual female adult mosquitoes expressing rtTA using the bZip or vasa promoters, following egg laying. The abundances are relative to a female mosquito from bZip-rtTA M1–2 line. (b) qRT-PCR done on cDNA from pools of eggs laid by 10 females from each group. The results represent the mean mRNA abundances, relative to bZip-rtTA M1–2 line ±SD, n=3. The data were analyzed by one-way ANOVA, * p≤0.05. In both qRT-PCR reactions the ribosomal S7 protein gene was used as the house keeping gene.

We selected 4 different lines originating from G_1_ female mosquitoes (2 from the M-group and 2 from the F-group) with the highest abundance of rtTA mRNA. We compared the rtTA mRNA levels of the 4 mosquito lines (M1F, M2i, F2C and F2e) to that of 3 transgenic lines we had previously created that express rtTA under the early embryonic bZip promoter, M1–2, M3–2 and M3–3. The rtTA mRNA level in bZip-rtTA M1–2 line was used as a reference. The rtTA mRNA abundance in all three bZip-rtTA lines were comparable (Fig. 2a). In the vasa-rtTA lines mRNA abundance was 1,538-, 2,326-, 562- and 380-fold higher in M1f, M2i, F2c and F2e lines, respectively than in reference bZip-rtTA line (M1–2) (Fig. 2a).

Line F2e was lost due to an insectary failure. We crossed the transgenic vasa-rtTA M1f, F2c and M2i lines to WT through 4 generations to expand the lines and reduce the number of inserts. Since the vasa promoter can drive expression in the early stages of embryonic development (57), we assessed rtTA transcript abundance in embryos 1 h after oviposition (Fig. 2b). The mean rtTA mRNA abundance was 1714-, 2,813- and 405-fold higher than in eggs from the bZip-rtTA line M1–2, in the eggs from the M1f, M2i and M1–2 lines respectively. As seen in the adult mosquitoes, the rtTA mRNA abundance in M2i was higher than in all other lines, and significantly higher compared to F2c embryos (Fig. 2a and b).

### Mapping the transposon-based insertions of the driver construct and generation of homozygous mosquitoes

From G_6_ onwards, the lines were in-crossed to obtain homozygosity of the insertion. We collected genomic (g)DNA at G_7_ and used it to identify the genomic locus of the transposon-based insertion in each line using the Splinkerette PCR protocol (see materials and methods). The sequences were aligned against the *An. Stephensi* genome in VectorBase. In the M1F line, the vasa construct was inserted into supercontig KB664888, position 665,462, which is within an exon in the open reading frame (ORF) of an “Era G-protein-like” (ASTE010067) coding sequence. In the F2c line the insertion was in supercontig KB665088, position 785,341, which is not in any known ORF. In the M2i line, the vasa construct was inserted into supercontig KB665343 position, 382,729, which is within the last intron of a putative juvenile hormone diol kinase gene (ASTE000415).

**Figure 3:**
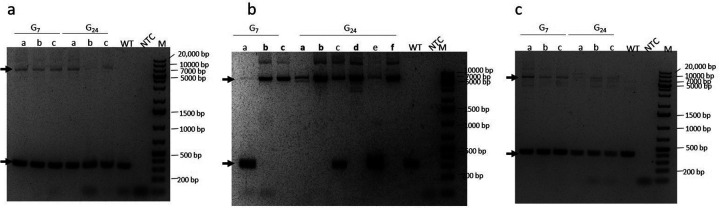
Diagnostic PCR to identify the zygosity status of the insertion in M1f (a), M2i (b) and F2c (c) driver lines. PCR was done on genomic DNA from 3 individual mosquitoes (a-c) from all 3 lines, collected at G7. At G24, mosquitoes (a-c) were collected for M1f and F2c and 6 were collected for M2i (a-f). Genomic DNA from wt. *A. stephensi female* mosquitoes was used as a negative control and water was used as a no template control (NTC). The expected PCR products for the M1F insertion and WT alleles were 7560 and 354 bp, respectively. For M2i the products of the insertion and WT alleles were 7534 and 328, respectively. The expected PCR product for the F2c insertion and WT alleles were 7636 and 430 bp, respectively. Black arrows indicate the position of the PCR products on the gel. Homozygous mosquitoes’ labels are bolded. Homozygous mosquitoes were found only in M2i.

We continued to in-cross the 3 lines through subsequent generations while monitoring the proportion of mBanana expressing larvae in each generation as an indication of their progression toward homozygosity. In the M2i line almost all the larvae were transgenic after G_17_, while in the F2c and M1f lines only about two thirds of the larvae expressed mBanana. We therefore carefully tracked the proportion of mBanana-positive larvae through generations 24, 25 and 26 (Tables 1). All M2i larvae expressed mBanana in generations 24–26. Only 76%, 74% and 80% of M1f larvae and 77%, 72% and 78% of F2c larvae expressed mBanana in generations 24, 25 and 26, respectively. Based on the positions of the inserts in each line, we designed PCR primers flanking the insertions to determine the zygosity level in each line (Fig. 3). All three M1f and F2c tested mosquitoes were heterozygous at G_7_ and continued to be heterozygous at G_24_. Two of 3 mosquitoes in the M2i line were homozygous at G_7_ (b and c), and 4 of 6 mosquitoes (a, b, d and f) were homozygous at G_24_ (Fig. 3).

### Assessment of potential insertion-related fitness cost

**Figure 4:**
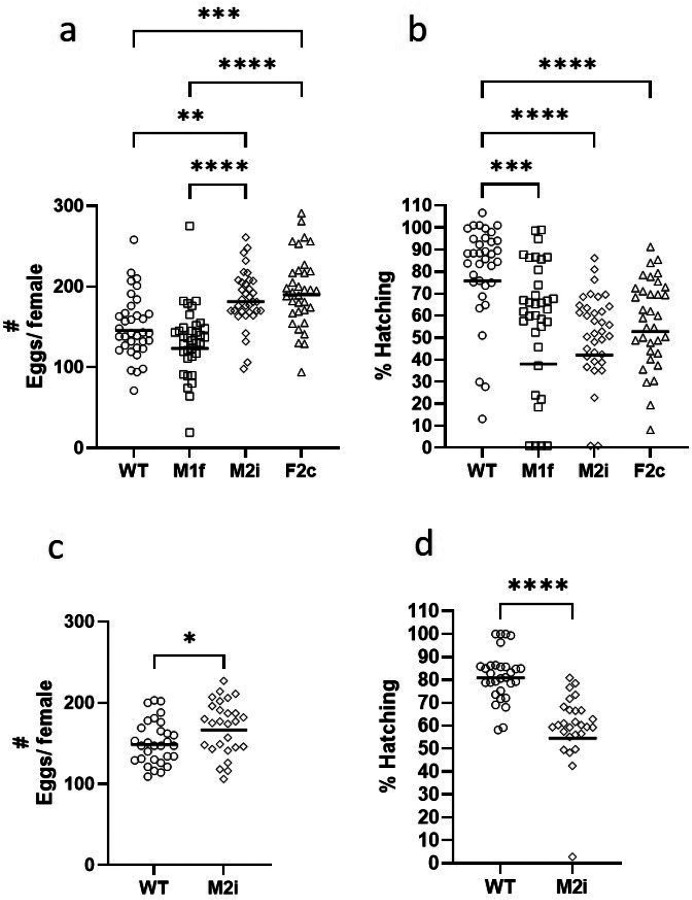
Fecundity in WT and driver line mosquitoes. (a) The number of eggs laid per individual female in *Drosophila* tubes in experiment #1. (b) The percentage of eggs hatching in experiment #1 was determined by counting the number of larvae in the *Drosophila* tubes one day after egg laying. In both panels the results are reported as the geometric means, n=34 for F2c and n=35 for WT, M1f, and M2i lines. In panel B, some values were zero. Whenever a value was 0, it was arbitrarily assigned a value of 1 to allow calculation of the geometric means. (c) The number of eggs laid per individual female in *Drosophila* tubes in experiment #2. (d) The percentage of eggs hatching in experiment #2.

To assess the effect of the rtTA expression on fitness, we compared the fecundity in all 3 lines with that of WT *An. stephensi*. From each line, eggs from up to 100 individual females were counted and the proportion of eggs hatching determined. From the WT line, 83% laid eggs. Likewise, 86% and 84% of the females laid eggs in the M2i and F2c groups, respectively. Only 59 out of 86 (68%) M1f females laid eggs. The geometric mean (GM) number of eggs laid per individual female in the WT line was 145, (95% CI = 133–159). In the M1f line the number of eggs/ female was slightly but not significantly lower [GM = 123, 95% CI = 106–143), while the numbers of eggs laid by M2i (GM = 181, 95% CI = 169–195) and F2c (GM = 190, 95% CI = 175–207) females were significantly higher than those of WT and the M1f line (Fig. 4a). The GM of the percentage larvae hatching in WT was 76% (95% CI = 65–88). Hatching rates were significantly lower in all three driver lines, 38% (95% CI = 45–63) in M1f, 42% (95% CI = 30–59) in M2i, and 53% (95% CI = 45–62) in F2c (Fig. 4b). In the following generation, the number of eggs laid per M2i female (GM = 167, 95% CI = 154–181) was again higher than WT females (GM = 149, 95% CI = 139–159) (Fig. 4c), and the hatching rate in M2i (GM = 55%, 95% CI = 44–70) was significantly lower compared to that of WT (GM = 81, 95% CI = 77–85) (Fig. 4d), confirming the pervious observation (Fig. 4 a&b).

## Discussion

Our long -term goal is to establish a system for conditionally expressing foreign genes in *An. stephensi*. Our medium-term goal is to use this system to conditionally express a lethal gene in male mosquitoes to increase the efficiency of our manufacturing process. In the present study we created the driver line for the conditional expression that can be crossed with the effector line to generate mosquitoes with the desired phenotype.

We evaluated two promoters for driving expression of rtTA. For unknown reasons, transcription of rtTA was minimal under bZip. This finding is consistent with a previous study done in *Ae. aegypti,* which showed that while the bZip promoter effectively drove strong transcription of the mNG fluorescent marker, it failed to initiate transcription of tTA (58). In contrast, the vasa promoter facilitated high levels of rtTA expression. The vasa promoter has been shown previously to drive endogenous vasa gene expression as well as exogenous eGFP expression in ovaries, testis and 1–2 h post-oviposition embryos of An. *gambiae* (57).

We were unable to obtain homozygosity in lines M1f and F2c, while in the 3rd line, M2i, homozygosity was established as early as G_7_. rtTA mRNA expression in M2i was higher than in M1f and F2c, likely due the fact that there was an rtTA encoding gene on both chromosomes, not just one. Thus, it appears that the inability to reach homozygosity in M1f and F2c was not due to rtTA expression but rather to the genomic locus of the insertions.

In the M1f line the rtTA insertion was within an exon in a coding gene, Era G-protein-like (ASTE010067), a member of a family of G-proteins associated with signal transduction and involved in many aspects of the life cycles in many organisms. One essential, well known function of G-proteins in insects, including Anophelines, is in the sensing of environmental signals through odorant binding proteins and the response of the insects to those signals (59, 60). It is therefore highly likely that ASTE010067 is an essential gene, and the mosquitoes cannot tolerate disruption of both of its copies. In F2c, the insertion was at the 3’ end of supercontig KB665088, in a non-coding region, which may be crucial for mosquito survival.

In M2i, the insertion was in an intron of a putative juvenile hormone diol kinase gene (ASTE000415). In the moth, *Heortia vitessoides,* juvenile hormone diol kinase is involved in hormone recycling and is essential for the development and survival of the moth (61). The ability to obtain homozygosity suggests that the insertion in the intron did not affect the expression and activity of the gene. Four out of 6 G_24_ M2i mosquitoes were homozygous for the insertion, indicating that the insertion was still not fixed in the population. It was therefore surprising that by microscope screening, 100% of the larvae were mBanana positive. It is possible that in M2i there are one or more insertions in genes in addition to the putative juvenile hormone diol kinase gene (ASTE000415) that were not detected by the Splinkerette mapping. The fact that all the larvae were positive by microscopy in three consecutive generations (G_24_-G_26_) suggests that at least one of the undetected insertions is already fixed in the population and its position is not in an essential locus in the genome.

The homozygous line, M2i produced significantly more eggs (24% and 12%) than the WT mosquitoes in two experiments (Fig. 4). However, M2i had a significantly lower (45% and 33%) rate of larval hatching in (Fig. 4). Thus, the efficiency overall of producing larvae was reduced by about 21%. Since the driver line will next be crossed with an effector line, it remains to be seen whether this reduced hatching rate will impact production of adults in the final product. Expression of tTA has been associated with fitness costs in insects. Overexpression in *D. melanogaster* had a toxic effect on early development in heterozygous progeny due to interference in ubiquitination and protein proteolysis as well as transcriptional squelching (41). Thus, it was used later as a lethal gene in RIDL systems in *Ae. aegypti* and *D. melanogaster* (40, 46). The lethal effect of tTAV, a codon-optimized version of tTA, on transcription in D *melanogaster* is dependent on the site of the genomic integration. All insertions have negative effects on early development, but each insertion triggers a different transcriptional profile leading to different phenotypes (40). Based on these observations, Oxitec developed transgenic mosquitoes in which females are being killed in the field by overexpressing the tTA in a positive feedback loop (46, 62, 63). In these mosquitoes, only minimal levels of tTA are being produced when tetracycline is present in the larval diet but when these mosquitoes are released in the field with no tetracycline, the tTA binds to TetO allowing overexpression of the downstream tTA in females, and mortality of subsequent mosquito generations at the early larval stage. Release of these mosquitoes in field studies in the United States and Brazil led to reductions of up to 96% of the mosquito populations (47, 50).

## Conclusions

In summary, we successfully completed the first step in creating *An. Stephensi* with a male-lethal allele controlled by a conditional gene expression system. We produced *An. stephensi* homozygous driver line, M2i, that expresses rtTA under the early embryonic vasa promoter. Egg production by m2i was ~ 18% greater than by WT mosquitoes, and the hatching rate of eggs to larvae ~ 39% lower than in WT mosquitoes. This overall 21% decrease in fecundity is acceptable for moving to the next step which will be to cross the M2i driver line with an effector line carrying a male-lethal gene. In the final product the rtTA transcription factor will bind to the tetracycline responsive element (TRE) in the presence of doxycycline, driving lethal gene expression only in males. Success in step 2 will provide the foundation for crossing the driver line described herein with other lines to conditionally express any target gene in *An. stephensi*.

## Figures and Tables

**Table 1:** Proportion of transgenic mosquitoes expressing the mBanana fluorescent protein after 24, 25 and 26 generations of selection. Only the M2i line reached homozygosity.

Line	Generation	mBanana Positive	mBanana Negative	Total	Proportion mBanana Positive
M1f	24	174	54	228	76%
	25	194	77	268	74%
	26	223	95	318	70%
M2i	24	235	0	235	100%
	25	264	0	264	100%
	26	323	0	323	100%
F2c	24	197	60	257	77%
	25	173	66	239	72%
	26	283	78	361	78%

## Data Availability

All data generated or analyzed during this study are included in this published article.
